# Chest computed tomography characteristics of critically ill COVID-19 patients with auto-antibodies against type I interferons

**DOI:** 10.21203/rs.3.rs-3029654/v1

**Published:** 2023-06-13

**Authors:** Baptiste Lafont Rapnouil, Youssef Zaarour, Romain Arrestier, Paul Bastard, Bastien Peiffer, Elsa Moncomble, Mélodie Parfait, Raphaël Bellaïche, Jean-Laurent Casanova, Armand Mekontso-Dessap, Sébastien Mule, Nicolas de Prost

**Affiliations:** Hôpital Henri Mondor: Hopital Henri Mondor

**Keywords:** thoracic imaging, tomodensitometry, COVID-19, critical care, anti-IFN-I antibodies

## Abstract

**Purpose:**

patients with auto-antibodies neutralizing type I interferons (anti-IFN auto-Abs) are at risk of severe forms of coronavirus disease 19 (COVID-19). The chest computed tomography (CT) scan characteristics of critically ill COVID-19 patients harboring these auto-Abs have never been reported.

**Methods:**

Bicentric ancillary study of the ANTICOV study (observational prospective cohort of severe COVID-19 patients admitted to the intensive care unit (ICU) for hypoxemic acute respiratory failure) on chest CT scan characteristics (severity score, parenchymal, pleural, vascular patterns). Anti-IFN auto-Abs were detected using a luciferase neutralization reporting assay. Imaging data were collected through independent blinded reading of two thoracic radiologists of chest CT studies performed at ICU admission (±72h). The primary outcome measure was the evaluation of severity by the total severity score (TSS) and the CT severity score (CTSS) according to the presence or absence of anti-IFN auto-Abs.

**Results:**

231 critically ill COVID-19 patients were included in the study (mean age 59.5±12.7 years; males 74.6%). Day 90 mortality was 29.5% (n=72/244). There was a trend towards more severe radiological lesions in patients with auto-IFN anti-Abs than in others, not reaching statistical significance (median CTSS 27.5 (21.0–34.8] versus 24.0 (19.0–30.0), p=0.052; median TSS 14.5 (10.2–17.0) versus 12.0 (9.0–15.0), p=0.070). The extra-parenchymal evaluation found no difference in the proportion of patients with pleural effusion, mediastinal lymphadenopathy or thymal abnormalities in the two populations. The prevalence of pulmonary embolism was not significantly different between groups (8.7% versus 5.3%, p=0.623, n=175).

**Conclusion:**

There was no significant difference in disease severity as evaluated by chest CT in severe COVID-19 patients admitted to the ICU for hypoxemic acute respiratory failure with or without anti-IFN auto-Abs.

## Introduction

Severe acute respiratory distress syndrome virus 2 (SARS-CoV-2) infection leads to a broad spectrum of manifestations with vast inter-individual variability, ranging from asymptomatic presentations to severe coronavirus disease 2019 (COVID-19)-associated acute respiratory distress syndrome (ARDS) requiring intensive care unit (ICU) admission in 5–10% of cases [1]. The protective role of type I interferons (IFNs) immunity during SARS-CoV-2 infection was documented by the observation of life-threatening COVID-19 pneumonia in patients with inborn errors of immunity affecting Toll-like receptor 3 (TLR3) or TLR7-dependent type I IFNs induction and amplification, in 1–5% of cases of critical COVID-19 pneumonia [2, 3]. Autoimmune phenocopy of inborn errors of type I IFN-dependent immunity were also shown to underlie life-threatening COVID-19 pneumonia. Circulating IgG auto-antibodies (Abs) neutralizing IFN-α2 and/or IFN-ω (10 ng/mL) were found in 10% of critical COVID-19 cases in an international cohort, as compared with 0% of mildly/asymptomatic cases and 0.3% of uninfected individuals [4]. Auto-Abs neutralizing type I IFN [4–6] are now established as risk factors of developing severe COVID-19 in the general population and have been repeatedly found to have a prevalence of around 10% among critically ill COVID-19 patients [7]. Surprisingly, in a recent multicenter study of critically ill COVID-19 patients the presence of auto-Abs neutralizing type I IFN was not associated with outcome [8]. Such clinical observation was consistent with the previous finding that critically ill patients exhibit a deficient type I IFN-stimulated gene (ISG) response in myeloid cells, whether they harbor auto-Abs or not [9, 10].

The chest computed tomography (CT) characteristics of critically ill COVID-19 patients with auto-Abs neutralizing type I IFN have, to the best of our knowledge, not been studied. Chest CT has been widely used as a diagnostic, prognostic, and phenotyping tool, as well as a detection method for pulmonary thromboses associated with COVID-19 [11, 12]. In this ancillary analysis of an observational prospective cohort study, we aimed at studying the chest CT scans of patients with or without auto-Abs neutralizing type I IFN.

## Methods

### Study design and participants

This is an ancillary study of the ANTICOV study [8], an observational prospective French multicenter study (NCT04733105), which included patients between March 31st 2020 and May 1st 2021. This ancillary study included patients from the medical and surgical ICUs of Henri Mondor Hospital (Créteil, France). Inclusion criteria were as follows: age ≥ 18 years, SARS-CoV-2 infection confirmed by a positive reverse transcriptase-polymerase chain reaction (RT-PCR), patient admitted in the ICU for acute respiratory failure (SpO2 ≤ 90% and need for supplemental oxygen or any kind of ventilator support), chest CT performed at ICU admission ± 72 hours. The study was approved by the Comité de Protection des Personnes Nord-Ouest IV (N° EudraCT/ID-RCB: 2020-A03009–30). Informed consent was obtained from all patients or their relatives.

Demographics, clinical and laboratory variables were recorded upon ICU admission and during ICU stay in the original cohort. Additional data pertaining to thoracic medical history and exposure to pneumotoxic drugs relevant to guide CT interpretation were retrospectively gathered from the patients’ medical files. The severity of the disease upon ICU admission was assessed using the World Health Organization (WHO) 10-point progression scale [13] and the sequential organ failure assessment (SOFA score) [14].

### Evaluation of anti-interferon auto-antibodies by luciferase reporter assays

Auto-Abs positivity was assessed on serum samples collected during the first week of ICU admission. The blocking activity of anti-IFN-α2 and anti-IFN-ω auto-Abs was determined with a reporter luciferase activity, as previously described [5]. Briefly, HEK293T cells were transfected with a plasmid containing the Firefly luciferase gene under the control of the human interferon stimulating response element (ISRE) promoter in the pGL4.45 backbone, and a plasmid constitutively expressing Renilla luciferase for normalization (pRL-SV40). Cells were transfected in the presence of the X-tremeGene9 transfection reagent (Sigma-Aldrich, ref. number 6365779001) for 24 hours. Cells in Dulbecco’s modified Eagle medium (DMEM, Thermo Fisher Scientific) supplemented with 2% fetal calf serum (FCS) and 10% healthy control or patient serum (after inactivation at 56°C, for 20 minutes) were either left unstimulated or were stimulated with IFN-α2 (Milteny Biotec, ref. number 130–108-984), IFN-ω (Merck, ref. number SRP3061), at 10 ng/mL or 100 pg/mL, or IFN-β (Milteny Biotech, ref. number: 130–107-888) at 10 ng/mL, for 16 hours at 37°C. Each sample was tested once for each cytokine and dose. Finally, cells were lysed for 20 minutes at room temperature and luciferase levels were measured with the Dual-Luciferase^®^ Reporter 1000 assay system (Promega, ref. number E1980), according to the manufacturer’s protocol. Luminescence intensity was measured with a VICTOR-X Multilabel Plate Reader (PerkinElmer Life Sciences, USA). Firefly luciferase activity values were normalized against Renilla luciferase activity values. These values were then normalized against the median induction level for non-neutralizing samples and expressed as a percentage. Samples were considered neutralizing if luciferase induction, normalized against Renilla luciferase activity, was below 15% of the median values for controls tested the same day. In this study, having any neutralizing auto Abs regardless of its specificity was considered as positive for anti IFN auto Abs.

### Computed tomography assessment

Protocol for Chest CT acquisition of COVID-19 patients was as follows: helicoidal volumic acquisition from pulmonary apex to iliac crest, on a Revolution (General Electric, USA) CT machine, after automatic adaptation of mAs and kV. CT pulmonary angiogram was performed if there was clinical suspicion of pulmonary embolism or for patients requiring > 3L/minute of oxygen supplementation, using a dual-energy CT (80 kV and 140 kV) acquisition protocol after injection of iodine contrast media (IOMERON 350, Bracco, Fr). Images with pulmonary filter were systematically reconstructed.

**Figure 1:**
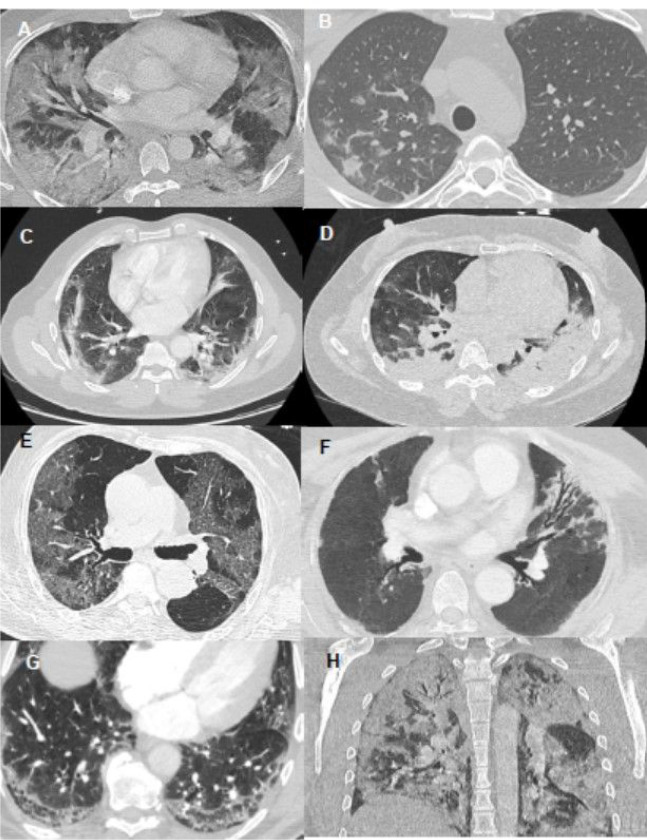
Representative panels of studied lung patterns. Axial views are shown except for the H panel. A. Mixed distribution (central and peripheral) hazy ground glass opacities; B. Pseudo-nodular ground glass opacities; C. Peripheral linear consolidations; D. Bilateral posterior condensed consolidations; E. Bilateral peripheral crazy paving; F. Bronchiectasis; G. Posterobasal honeycombing; H. Coronal view of both ground glass opacities and consolidations with a mixed distribution

Interpretation was performed independently by two radiologists with 5 and 8 years of experience in thoracic imaging (Y.Z and S.M.), blinded for anti-IFN Abs results. A systematic analysis was conducted based on a predefined reading grid. Representative examples of CT patterns are shown in Fig. 1.

Primary outcome measures : for severity assessment, we used two scores developed for COVID-19 patients, and based on semi-quantitative topographic evaluation of affected lung: the total severity score (TSS [15], cut-off value for severity 7.5) and the CT severity score (CTSS [16], cut-off value for severity 19). Discrepancies were resolved by a second reading by both radiologists.

### Statistics

Statistical analysis was performed using the R software (R Foundation for Statistical Computing, Vienna, Austria). A Kruskal-Wallis test was used to compare continuous variables and a Chi^2^ test was used for categorical variables (or a Fischer exact test when Chi^2^ requirements were not met). Two-tailed p-values <0.05 were considered statistically significant.

## Results

### Clinical characteristics and outcomes of the patients

**Figure 2:**
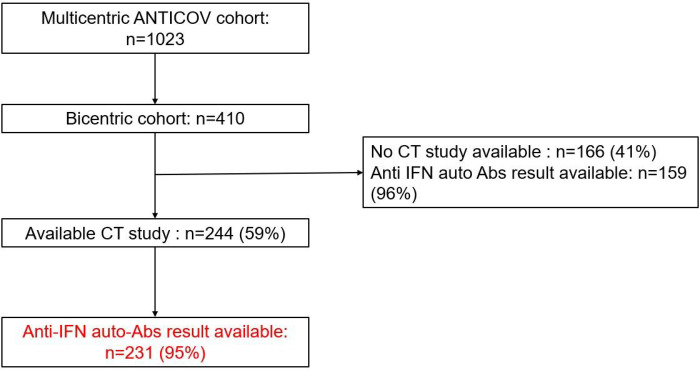
Flowchart of included patients

During the study period, there were 410 patients admitted in the ICUs at Henri Mondor Hospital who met inclusion criteria for the ANTICOV study. Among them, 244 (59%) patients had a chest CT scan (175 (76%) with pulmonary angiogram) obtained in the 72h time frame around ICU admission, including 231 patients (95%) who had anti-IFN auto-Abs results available, and who constituted the study population (Fig. 2).

Population characteristics are shown in Table 1. Most common comorbidities were hypertension and diabetes in 109 (44.7%) and 69 (28.3%) patients, respectively. Immunosuppression and history of relevant intrathoracic disease were found in less than 5% of patients. Median time (interquartile range, IQR) between first symptoms and admission was 9 (6–12) days, and WHO severity scale was 6 (6–8). Thirty patients (13%) had a positive result for anti-IFN auto-Abs, consistent with previous findings [4]. Day 90 mortality was 29.5% (n = 72/244).

Comparison between patients with or without a chest CT study available yielded no significant difference for most of demographic and admission characteristics (**Table S1**, online supplement). However, there were statistically significant differences regarding the frequency of several comorbidities (i.e., body mass index, asthma, hypertension, history of intrathoracic neoplasia) possibly related to COVID-19 severity at admission, with also higher WHO severity scale, and SOFA scores in patients who had no chest CT scan performed than in others. There was also a higher proportion of patients receiving ECMO in patients without a chest-CT, as expected given the risks of performing CT scan in unstable patients (**Table S1**).

### Chest CT scan patterns of critically ill COVID-19 patients

CT characteristics of severe COVID-19 patients displayed bilateral opacities in 99.1% of cases (n = 229/244), with ground glass opacities in 97.4% (n = 225/244) of them (Table 2). Severity scores were high with a median (IQR) CTSS of 24.0 (19.0–30.0) and TSS of 12.0 (9.0–15.0). Consolidations and interstitial changes were found in 80.5% (n = 186/244) and 22.5% (n = 52/244) of patients, respectively. The most frequent interstitial change was bronchiectasis in 20.3% (n = 47/244) of patients. Finally, the prevalence of pulmonary embolism in the subset of patients who had a CT pulmonary angiogram study performed was 5.7% (n = 10/175).

### Chest CT scan patterns of critically ill COVID-19 patients with positive anti-IFN auto-Abs

Regarding severity scores, there was a trend towards a more severe disease in patients with auto-IFN anti-Abs than in others, though not reaching statistical significance (median CTSS 27.5 (21.0–34.8] versus 24.0 (19.0–30.0), p = 0.052; median TSS 14.5 (10.2–17.0) versus 12.0 (9.0–15.0), p = 0.070). Comparison of the two groups (Table 2) showed no statistically significant difference for ground glass opacities (96.7% versus 97.5%, p0.570), its distribution, or its aspect. Similarly, there was no difference in terms of prevalence or aspect of parenchymal interstitial changes. Alveolar infiltrates were observed in similar proportions in patients with or without anti-IFN auto-Abs (73.3% vs 81.6%, p = 0.323), but with a pattern distribution being statistically different between the two populations (6.7% versus 27.9% of linear and 66.7 versus 54.2% of condensed consolidation, p = 0.025 for pattern distribution comparison). The extra-parenchymal evaluation found no difference in the proportion of patients with or without pleural effusion (23.3% versus 17.4%, p = 0.449), lymphadenopathy (50% versus 42.3%, p = 0.437) or pulmonary embolism prevalence between the two populations (8.7% versus 5.3%, p = 0.623, n = 175). The prevalence of thymic abmormalities was not significantly different between groups (26.7% versus 19.9%, p = 0.468). Because thymus might be involved in auto-immunity processes, we further explored the clinical characteristics and outcomes of the 8 patients with thymic abmormalities and auto-IFN anti-Abs (Table 3) and found no patient who developed a thymoma or a myasthenia during follow-up. Yet, one additional patient, who could not be included in the cohort because he had a chest CT scan performed outside the predefined time window (i.e., 6 days before ICU admission), presented a histologically-confirmed thymoma (Table 3).

## Discussion

During the pandemic, the search for risks factors of severe forms of COVID-19 beyond the rapidly described demographic and clinical factors such as age, sex, hypertension and overweight, has led to the identification of innate or acquired genetic or immunological predispositions [2, 4]. Among those, the presence of circulating anti-IFN auto-Abs has been demonstrated to be associated with a higher risk of severe COVID-19 and mortality in the general population and in patients with mild disease [6, 17]. This was consistent with the identification of inborn errors of TLR3- or TLR7-dependent type I IFN immunity [2, 3, 18–20]. However, their impact on mortality is less clear in COVID-19 patients already admitted in the ICU [8]. To our knowledge, this is the first study of thoracic imaging of patients with anti-IFN auto-Abs admitted in the ICU for severe COVID-19.

Patients with anti-IFN auto-Abs showed a non-significant trend towards more severe and extensive lesions during the early phase of COVID-19-associated acute respiratory failure. Alveolar infiltrates were more frequently condensed than linear, possibly reflecting a more severe disease. These findings are consistent with the literature given the association, on the first hand, between these antibodies, the severity of COVID-19 [6] and oxygen supplementation requirement at ICU admission [8], and, on the other hand, between the extent of pulmonary infiltrate on chest CT and disease severity [12]. Indeed, we could expect pulmonary inflammation to be exacerbated in anti-IFN auto-Abs patients who are lacking competent innate immunity to control viral replication [21]. However, ground glass opacities, which have been shown to be a surrogate of inflammatory lung injury [22], were not different between groups. This finding is in line with a recent study of severe COVID-19 patients showing no significant different broncho-alveolar fluid concentration of inflammatory markers in patients harboring anti-IFN auto-Abs than in those who did not [23]. These results are consistent with the hypothesis of a common pathogenesis for critical COVID-19 involving impaired type I IFN responses in all patients, as suggested by the similarities between patients with and without anti-IFN auto-Abs [10], and are in line with the previously suggested hypothesis of a global emerging framework of an IFN I deficiency causal for critical COVID-19, where anti-IFN auto-Abs are only one of the known and unknown mechanisms for this deficiency [19].

There was also no significant difference in the prevalence of pulmonary embolism (PE) in patients with versus without anti-IFN auto-Abs. The following factors should however be taken into account to interpret these findings: (1) CT pulmonary angiograms were not routinely performed, especially in the more severe patients potentially leading to a selection bias; (2) The overall 5.7% PE rate in our cohort was lower than that reported in historical series (23.3–29.6% in a meta-analysis [11]) reducing our statistical power and our ability to detect between-group differences. We did not find specific data on anti-IFN auto-Abs and thrombosis during severe COVID-19 but the relationship between innate immunity, inflammation and coagulation has been well described [24], and might be specifically relevant given the endothelial tropism of SARS-CoV-2 [25].

The origin of anti-IFN auto-Abs remains unknown but there is a hypothesis about the role of the thymus in their genesis [17, 26]. This hypothesis is backed up by observations in other human diseases, such as the link between auto-antibodies-mediated myasthenia gravis and thymoma [27]. Of note, most of the patients with autoimmune polyendocrine syndrome type 1 (APS-1), a disease characterized by the loss of thymic central immune-tolerance, carry anti-IFN auto-Abs [28]; these patients are at risk for severe COVID-19 [4]. Moreover, the loss of immune-tolerance accompanying thymic aging [29] might account for the increasing prevalence of anti-IFN auto-Abs with age [5]. In our study, however, there was no significant difference regarding the presence of thymic abnormalities between patients having anti-IFN auto-Abs or not, a finding that might argue against thymic involvement in the genesis of these auto-Abs. While none of 8 reported patients included in the cohort developed thymoma at latest known follow-up, we report one additional patient who developed a histologically confirmed thymoma, suggesting that in rare instances there might be an association between thymoma and anti-IFN auto-Abs.

Our study has several limitations. First, it included only two centers, making it susceptible to selection biases (tertiary care centers) and different care practices limiting its external validity. However, chest CT scans analyzed in the current study were obtained early in the hospital stay so that the impact of management strategies on CT scan patterns was probably low. Including the patients from the whole cohort might have helped generalizing the results but would have implied transferring CT scan images from other centers. Second, the ancillary part of this study was retrospective and CT studies were not performed routinely in all patients. Populations of patients with and without CT data available were largely comparable, suggesting the risk of selection bias is low. Third, the 72-hour time frame around ICU admission for CT acquisition is debatable, as COVID-19 is well known for its dynamic evolution in two phases (replicative and inflammatory) [21]. Because a longitudinal follow-up was impossible, this time window was arbitrarily chosen to capture the early phase of severe SARS-CoV-2 infection and avoid the risks of chest CT pattern changes attributable to the ICU stay.

Our study also has strengths, including the relatively large number of critically ill patients included, having been screened for anti-IFN auto-Abs and chest CT scan, and the independent blinded interpretation protocol of chest CT scan by chest expert radiologists.

In conclusion, the presence of anti-IFN auto-Abs in critically ill patients with severe COVID-19 was not associated with significant differences in chest CT severity scores.

## Figures and Tables

**Table 1:** Characteristics of patients with available chest computed tomography scan included in the study.

	Study population n = 244
Age, years	59.5 (12.7)
Male	182 (74.6)
BMI, kg/m^2^	29.3 (5.6)
Clinical frailty scale	3.0 (2.0–3.0)
Comorbidities	
Diabetes	69 (28.3)
Hypertension	109 (44.7)
Chronic cardiac failure	31 (12.7)
Chronic kidney disease	21 (8.6)
Cirrhosis	3 (1.2)
COPD / Chronic respiratory failure	14 (5.7)
Asthma	10 (4.1)
Immunosuppression	
HIV infection	3 (1.2)
Solid organ transplant	12 (4.9)
Hematological malignancy	3 (1.2)
Chronic corticosteroid treatment	14 (5.7)
Auto-immune disease	9 (3.7)
History of intrathoracic disease	
Pneumonia	9 (3.7)
Tuberculosis	2 (0.8)
Pleural effusion	4 (1.6)
Neoplasia	2 (0.8)
Cervical or thoracic radiotherapy	4 (1.6)
Pneumotoxic drug exposure	28 (11.5)
Admission data	
Days between symptoms and ICU admission	9.0 (6.0–12.0)
WHO severity scale	6.0 (6.0–8.0)
Dexamethasone for COVID-19	128 (52.5)
Tocilizumab	22 (9.0)
ECMO	2 (0.8)
SOFA	3.0 (2.0–4.0)
Anti-IFN auto-Abs positivity	30 (13.0)
Outcome	
Deceased at day 28	57 (23.4)
Deceased at day 90	72 (29.5)

Results are N (%), mean (± standard deviation) or median (interquartile range); CT: computed tomography; SD: standard deviation; BMI: body mass index; COPD: chronic obstructive pulmonary disease; HIV: human immunodeficiency virus; ICU: intensive care unit; WHO: World Health Organization; COVID-19: coronavirus disease 19; ECMO: extracorporeal membrane oxygenation; SOFA: sequential organ failure assessment; IFN: interferon; Abs: antibodies

**Table 2:** Chest CT features of patients with or without anti-interferon auto-antibodies.

	Anti IFN auto Abs n = 30	No anti IFN auto Abs n = 201	P value
Bilateral opacities	30 (100.0)	199 (99.0)	1.000
Basal predominant distribution	9 (30.0)	49 (24.4)	0.504
Pleural and parenchymal analysis			
Ground glass opacities	29 (96.7)	196 (97.5)	0.570
Distribution			0.533
Peripheral	12 (40.0)	97 (48.3)	
Central	0 (0.0)	0 (0.0)	
Mixed	17 (56.7)	99 (49.3)	
Aspect			0.829
Pseudo nodular	1 (3.3)	7 (3.5)	
Hazy	28 (93.3)	189 (94.0)	
Crazy paving	10 (33.3)	69 (34.3)	1.000
Alveolar infiltrates	22 (73.3)	164 (81.6)	0.323
Aspect			0.025
Linear	2 (6.7)	56 (27.9)	
Condensed	20 (66.7)	109 (54.2)	
Lobar pneumonia	1 (3.3)	30 (14.9)	0.091
Interstitial changes	5 (16.7)	47 (23.4)	0.490
Aspect			0.386
Bronchiectasis	4 (13.3)	43 (21.4)	
Septal thickening	0 (0.0)	1 (0.5)	
Fibrous bands	0 (0.0)	2 (1.0)	
Honeycombing	1 (3.3)	1 (0.5)	
Micronodules	1 (3.3)	3 (1.5)	0.429
Emphysema	5 (16.7)	33 (16.4)	1.000
Pleural effusion	7 (23.3)	35 (17.4)	0.449
Mediastinal lymphadenopathy	15 (50.0)	85 (42.3)	0.437
Thymic abnormality	8 (26.7)	40 (19.9)	0.468
Pulmonary thrombosis†	2 (8.7)	8 (5.3)	0.623
Severity scores			
CTSS (median, IQR)	27.5 (21.0–34.8)	24.0 (19.0–30.0)	0.052
TSS (median, IQR)	14.5 (10.2–17.0)	12.0 (9.0–15.0)	0.070

Results are N (%), means (± standard deviation) or median (interquartile range); IFN: interferon; Abs: antibodies; CTSS: computed tomography severity score; TSS: total severity score; IQR; interquartile range.

† n = 175 patients with available pulmonary angiography.

**Table 3:** Characteristics of patients with abnormal thymus on chest CT scan and positive anti-IFN auto-Abs

Age	Gender	Medical history	Thymic finding	Anti-IFN auto-Ab	Follow-up*
**Characteristics of the 8 patients with both abnormal thymus *on chest CT scan* and positive anti-IFN auto-Abs included in the cohort**					
64	M	No significant history	Nodular	α2 10ng/mL ω 10ng/mL	Deceased
69	M	Diabetes Hypertension Smoking	Non specific	α2 100pg/mL β 10ng/mL	No clinical or scannographic evidence for thymoma at 38 months
79	M	Diabetes	Non specific	α2 10ng/mL ω 10ng/mL	Deceased
41	F	Hypertension Lupus	Hyperplasia	α2 10ng/mL β 10ng/mL ω 100pg/mL	No clinical or scannographic evidence for thymoma at 37 months
28	F	Heart transplant Chronic heart failure	Non specific	α2 100pg/mL β 10ng/mL	Deceased
33	M	Hypertension Chronic kidney disease	Non specific	α2 100pg/mL β 10ng/mL	No clinical or scannographic evidence for thymoma at 39 months
64	M	Artery disease	Non specific	α2 100pg/mL ω 100pg/mL	No clinical or scannographic evidence for thymoma at 38 months
**Characteristics of the 8 patients with both abnormal thymus *on chest CT scan* and positive anti-IFN auto-Abs included in the cohort**					
67	F	Diabetes Hypertension	Non specific	ω 100pg/mL	Deceased
**Characteristics of an additional patient with both abnormal thymuson chest CT scanand positive anti-IFN auto-Abs not included in the cohort** **					
60	M	None	Thymal mass 40 × 50 mm	α2 10ng/mL ω 10ng/mL	Surgery 8 months after ICU discharge confirming AB thymoma stage pT1a No associated myasthenia

* Follow-up is based on a retrospective consultation of our institution medical record, no systematic or dedicated follow up protocol was used

** Initial chest CT scan was performed outside the ICU admission ±72 h time window for study inclusion (i.e., at hospital admission, 6 days before ICU admission); M: male; F: female

## Data Availability

Original data presented in the manuscript are available on reasonable request to the corresponding author.

## Abbreviations

Ab / Abs: antibody / antibodies
ARDS: acute respiratory distress syndrome
COVID-19: coronavirus disease 2019
CT: computed tomography
ICU: intensive care unit
IFN: interferon
IQR: interquartile range
SARS-CoV-2: severe acute respiratory syndrome coronavirus 2
SD: standard deviation
